# Accidents due to falls from roof slabs

**DOI:** 10.1590/1516-3180.2013.1313479

**Published:** 2013-06-01

**Authors:** Bruno Alves Rudelli, Marcelo Valério Alabarce da Silva, Miguel Akkari, Claudio Santili

**Affiliations:** I IResident. Department of Orthopedics and Traumatology, Hospital da Clínicas (HC), Faculdade de Medicina da Universidade de São Paulo (FMUSP), São Paulo, Brazil.; II MD. Resident in Sports Medicine, Universidade Federal de São Paulo (Unifesp), São Paulo. Brazil.; III MD, PhD. Professor in the School of Medical Sciences and Head of the Pediatric Orthopedic Group, Irmandade da Santa Casa de Misericórdia de São Paulo, São Paulo, Brazil.; IV MD, PhD. Adjunct Professor in the School of Medical Sciences and Attending Physician in the Pediatric Orthopedic Group, Irmandade da Santa Casa de Misericórdia de São Paulo, São Paulo, Brazil. Adisseo do Brasil Brazil

**Keywords:** Accidental falls, Child, Adolescent, Public health, Accident prevention, Acidentes por queda, Criança, Adolescente, Saúde pública, Prevenção de acidentes

## Abstract

**CONTEXT AND OBJECTIVE:**

Falls from the roof slabs of houses are accidents of high potential severity that occur in large Brazilian cities and often affect children and adolescents. The aims of this study were to characterize the factors that predispose towards this type of fall involving children and adolescents, quantify the severity of associated lesions and suggest preventive measures.

**DESIGN AND SETTING:**

Descriptive observational prospective longitudinal study in two hospitals in the metropolitan region of São Paulo.

**METHODS:**

Data were collected from 29 cases of falls from roof slabs involving children and adolescents between October 2008 and October 2009.

**RESULTS:**

Cases involving males were more prevalent, accounting for 84%. The predominant age group was schoolchildren (7 to 12 years old; 44%). Leisure activities were most frequently being practiced on the roof slab at the time of the fall (86%), and flying a kite was the most prevalent game (37.9%). In 72% of the cases, the children were unaccompanied by an adult responsible for them. Severe conditions such as multiple trauma and traumatic brain injuries resulted from 79% of the accidents.

**CONCLUSION:**

Falls from roof slabs are accidents of high potential severity, and preventive measures aimed towards informing parents and guardians about the dangers and risk factors associated with this type of accident are needed, along with physical protective measures, such as low walls around the slab and gates with locks to restrict free access to these places.

## RESUMO

**CONTEXTO E OBJETIVO:**

A queda de cima da laje é um acidente com alto potencial, que ocorre nas grandes metrópoles brasileiras e frequentemente acomete crianças e adolescentes. O objetivo do estudo foi caracterizar os fatores que predispõem às quedas da laje envolvendo crianças e adolescentes, quantificar a gravidade das lesões associadas e sugerir medidas preventivas.

**TIPO DE ESTUDO E LOCAL:**

Estudo prospectivo longitudinal observacional de caráter descritivo realizado em dois hospitais na região metropolitana de São Paulo.

**MÉTODOS:**

Foram coletados dados de 29 casos de queda da laje envolvendo crianças e adolescentes no período de outubro de 2008 a outubro de 2009.

**RESULTADOS:**

O gênero masculino foi o mais prevalente, com 84% dos casos. A faixa etária predominante foi a escolar (7 a 12 anos) com 44%. O lazer foi a atividade mais frequente praticada na laje no momento da queda (86%), sendo a brincadeira de empinar pipa a mais prevalente (37,9%). Em 72% dos casos, as crianças estavam desacompanhadas de um adulto responsável. Quadros graves como politraumas e traumas cranioencefálicos representaram 79% dos acidentes.

**CONCLUSÃO:**

A queda da laje representa acidente com alto potencial de gravidade, sendo necessária a adoção de medidas preventivas direcionadas ao esclarecimento de pais e responsáveis sobre os perigos e fatores de risco relacionados com esse tipo de acidente, além da adoção de medidas de proteção física, como muretas ao redor da laje e portões com travas para restringir o livre acesso a esse local.

## INTRODUCTION

Injuries from external causes are the leading cause of death among people between 1 and 40 years of age in Brazil^1^ and in the city of São Paulo.^2^ External causes include the concepts of accident and violence (whether incidental or not), and are defined as trauma, injury or any other health problems, whether intentional or not, which have a sudden onset and are a direct result of violence, poisoning, car accidents or other exogenous causes.^3^ In 2009, accidental falls were the second biggest cause of deaths from external causes, second only to homicides in São Paulo.^2^

Falls are the commonest trauma mechanism among children and adolescents in Brazil^4^^,^^5^^,^^6^^,^^7^^,^^8^ and in the world.^9^^,^^10^ They may cause serious clinical conditions, with a high risk of complications.^4^^,^^11^^,^^12^ In Brazil, in metropolitan areas like São Paulo, a peculiar kind of fall from height exists: falls from the concrete slabs that form the roofs of houses. ^1^^,^^4^^,^^7^^,^^13^ This phenomenon is due to the presence of numerous shantytowns, which in the city of São Paulo have come to account for 7% of total urban settlements.^14^ Shantytowns are forms of urban settlement used by the low-income population, in which dwellings are built so crowded together that they barely offer space for recreation, either inside or outside the home. Often, due to economic difficulty, the construction remains unfinished, and the home is covered only with a concrete roof slab. Lack of other space makes the roof slab a site for a variety of activities, and thus favors this type of accident.

Falls from roof slabs present a serious risk to the victim’s physical integrity because of the high potential for severe injuries resulting from this type of accident.^1^^,^^4^^,^^7^^,^^12^^,^^13^ This applied especially to the age group of children and adolescents, because these individuals are more exposed to this type of trauma. In the scientific literature, there is a lack of studies characterizing this type of accident and promoting measures to prevent these falls.

## OBJECTIVE

The objectives of this study were to characterize the factors that predispose towards falls from roof slabs involving children and adolescents living in a specific area on the outskirts of São Paulo, to quantify the severity of associated lesions and, based on that knowledge, to suggest preventive measures that can be applied to the population at risk.

## METHODS

The study design was longitudinal, observational and prospective, and was conducted between October 2008 and October 2009. Over that period, 50 patients who had suffered falls from roof slabs were admitted to the emergency rooms of Hospital São Luis Gonzaga (HSLG) and General Hospital of Guarulhos (HGG). General Hospital of Guarulhos is the referral hospital for cases of medium and high complexity for 15% of the population living in the metropolitan region of São Paulo, and had approximately 122,000 emergency room visits in 2011. Hospital São Luis Gonzaga provides care for the north part of São Paulo and had 253,000 emergency room visits in 2011. Both hospitals are an important part of the healthcare system in the metropolitan region of São Paulo. Most of this region registers frequent occurrences of falls from roof slabs.

The cases were recorded using a protocol that asked for the following information: name, gender, telephone number, date of accident, age, diagnosis, medical management, region of the city where the fall occurred, the activity that was being practiced on the roof slab at the time of the fall and, if the patient was a child or adolescent, whether an adult had been with the patient at the time of the fall. This protocol was attached to the patient’s chart by the MRSS (Medical Records and Statistics Service) at the reception of the emergency room and was completed by the doctor on duty who treated these patients.

When the protocol was not completed by the doctor on duty, the MRSS official separated the medical records and the academic researcher collected the necessary data directly from the patient or a family member. After the information had been gathered, only the cases of patients younger than 18 years of age were selected and included in this study.

Over the study period, there were 50 cases of falls from roof slabs, 29 involving children and adolescents. The data were presented in tables and were analyzed using descriptive statistical methods.

## RESULTS

Among the 50 cases of falls from roof slabs, 29 (58.0%) were children or adolescents (0-18 years), and there was higher prevalence of cases among males, with 24 cases (82.8%).

**Graph 1.**
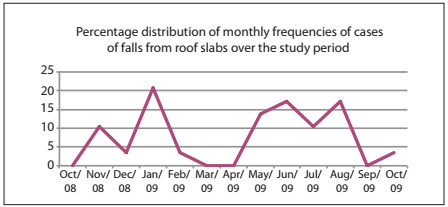
Percentage distribution of monthly frequencies of cases of falls from roof slabs over the study period.

The age distribution is shown in Table 1. The months with the highest incidence of this sort of accident were: January, representing six cases (20.7%); May, four cases (13.8%); June, five cases (17.3%); and August, five cases (17.3%). The distribution of all 29 cases by months of occurrence is shown in Graph 1.

**Table 1.** Distribution of the falls from roof slabs according to the age groups of the children and adolescents seen in the two hospitals, between October 2008 and October 2009

Age Group	Frequency	%
Preschool (0 to 6 years)	9	31
School children (7 to 12 years)	13	44.8
Adolescents (13 to 18 years)	7	24.2
Total	29	100

Most accidents occurred in the afternoon (12:01 to 18:00 hours), accounting for 22 cases (72.8%). Mondays were the day of the week with highest numbers, with nine cases (31.0%), but there was no statistical significance regarding the differences between days of the week.

Among the activities practiced on the roof slab by children and adolescents at the time of the fall, leisure activities were in first place, with 25 cases (86.2%), followed by domestic chores with one case (3.4%). The other three cases (10.4%) were not determined. Among the children who were practicing leisure activities, 11 (37.9%) were flying a kite, 12 (41.4%) were playing other games, in one case (3.4%) there was a birthday party on the roof and in five cases it was not possible to obtain specific information.

In 21 cases (72.4%), the children or adolescents were not accompanied by an adult responsible for them at the time of the fall. The severity of the injuries is shown in Table 2.

**Table 2.** Severity of injury due to falling from a concrete roof slab among the cases treated at two hospitals between October 2008 and October 2009

Diagnosis	Frequency	%
Bruises/mild injuries	3	10.3
Fracture	2	6.9
Dislocation	1	3.4
Multiple trauma	3	10.3
Multiple trauma + traumatic brain injury	2	6.9
Traumatic brain injury	18	62.1
Total	29	100

## DISCUSSION

The metropolitan region of São Paulo contains a large number of shantytown (*favela*) developments, which are defined as clusters of homes without regular allotment of land, without official determination of streets and sidewalks, with varying distances between dwellings, where part of the building is constructed with adapted material and the remainder with masonry or other suitable material^15^ and each habitation unit has a small internal area. The small spaces available for leisure activities, along with the problem of public safety at these sites, cause roof slabs to become an alternative entertainment area, especially for children and adolescents who have virtually no choice of place to spend their free time when they are not in school. This can be demonstrated by the higher frequency of this kind of fall among school-age children and adolescents that occurred during the period when they were not at school, in January (summer vacation).

The falls from roof slabs occurred more frequently among males (82.8%). This finding is consistent with studies in the literature, which have indicated that boys form a group that is more susceptible to this kind of fall, just as they are in relation to other types of falls and accidents in general during childhood.^1^^,^^4^^,^^5^^,^^6^^,^^8^^,^^9^^,^^10^^,^^12^^,^^13^^,^^16^^,^^17^^,^^18^^,^^19^ There are many explanations for the higher frequency with which males are involved in accidents, especially when they are children and adolescents. According to Filocomo et al. and Spider et al., boys are exposed to activities that are more dynamic and achieve sociocultural freedom earlier than girls do,^6^^,^^13^ which might be a reason for greater involvement, although today there is greater stimulus towards exposure of girls to active recreational activities and sports.

Another condition clearly associated with falls from roof slabs was the fact that in 72.4% of the accidents, the children or adolescents were unaccompanied by their parents or any other adult responsible for them. This finding confirms the strong relationship of this type of accident with lack of supervision, which may mean that these accidents could be avoided through the presence of a responsible adult, since the child would be guided towards preventive protective measures. On the other hand, there are some authors who disagree that the presence of a responsible adult could prevent this type of accident.^1^^,^^4^^,^^6^ Nonetheless, our view is that adults should be aware that their presence is important in relation to preventing these accidents.

Among the activities that were being practiced at the time of the fall, it was observed that flying a kite was very common. This is probably because the child’s attention was focused towards the sky, during an activity that requires movement and displacement in a small area that, in most cases, does not have any protection. Another aggravating factor is that the roof slabs are often close to electrical wiring, and therefore, besides the risk of falling, there is the risk of severe burns.

We did not find any significant variation in the accident rate according to the days of the week, even comparing weekdays and weekends. A difference between weekdays and weekends was expected, since children and adolescents have more free time to practice leisure activities during weekends.

Regarding the severity of the injuries resulting from these falls, it was found that 79.3% consisted of severe lesions, such as multiple trauma and traumatic brain injury, which can cause great social impact and enormous damage to the child’s health. Other authors have pointed out the importance of the relationship between falls from roof slabs and serious injuries.^7^^,^^13^^,^^16^^,^^18^ Campos et al.^7^ found that falls from concrete roof slabs were the main mechanism for spinal cord injuries among people under the age of 20 years, as well as the main mechanism for trauma affecting the cervical spine at any age. Gonçalves et al.^16^ also presented data that placed this type of fall as the primary cause of spinal cord injuries, representing 25% of all causes.

Regarding our sample, we know that the number of falls from roof slabs is certainly much higher than what is reported in this study. The small number included in the present report was due to the difficulty in obtaining data, partly because the attending physicians working in the emergency rooms only categorized this type of accident as falls from a height, and also because falls from roof slabs that are less severe are treated in hospitals that are not reference centers for complex cases, whereas the two hospitals that participated in this study have reference center status. Furthermore, in cases of death at the scene of the accident, the bodies are taken directly to the Death Verification Service.

Given these findings, it needs to be taken into consideration that most of these accidents could be avoided if parents, guardians and children were better oriented and if some physical barriers were put in place on roof slabs, such as protective walls and restrictions on access to the roof slab by means of some kind of obstacle, like a gate, which would depend on an adult for allowing its release.

## CONCLUSION

Falls from concrete roof slabs have a great impact on children and adolescents, since they lead to severe injuries (79.3% of the cases presented multiple trauma and head injury). Some factors are common to these accidents, such as practicing leisure activities, especially flying a kite, and lack of supervision by a responsible adult.

Preventive measures aimed towards informing parents and guardians about the dangers and risk factors associated with this type of accident should be encouraged in the communities at greatest risk. Physical protection measures such as protective walls or fences around roof slab and gates with locks to restrict free access to the roof slab are necessary in order to reduce the risk of such accidents.
